# An optimised dosing regimen versus a standard dosing regimen of vancomycin for the treatment of late onset sepsis due to Gram-positive microorganisms in neonates and infants aged less than 90 days (NeoVanc): study protocol for a randomised controlled trial

**DOI:** 10.1186/s13063-020-4184-8

**Published:** 2020-04-15

**Authors:** Louise F. Hill, Mark A. Turner, Irja Lutsar, Paul T. Heath, Pollyanna Hardy, Louise Linsell, Evelyne Jacqz-Aigrain, Emmanuel Roilides, Mike Sharland, for the NeoVanc Consortium, Carlo Giaquinto, Davide Bilardi, Mike Sharland, Paul T. Heath, Louise F. Hill, Timothy Planche, Tatiana Munera Huertas, Mark A. Turner, William Hope, Irja Lutsar, Emmanuel Roilides, Evelyne Jacqz-Aigrain, Wei Zhao, Louise Linsell, Peter Ghazal, Andrea Dotta, Javier de la Cruz, Clara Alonso Díaz, Susan Conroy, Louise Rawcliffe, Donato Bonifazi, Cristina Manfredi, Mariagrazia Felisi

**Affiliations:** 1 Paediatric Infectious Diseases Research Group, Institute for Infection & Immunity, St George’s, University of London, London, UK; 2 UCL Great Ormond Street Institute of Child Health, London, UK; 3 Institute of Translational Medicine, University of Liverpool, Liverpool, UK; 4 University of Tartu, Tartu, Estonia; 5 Birmingham Clinical Trials Unit, University of Birmingham, Birmingham, UK; 6 National Perinatal Epidemiology Unit, Oxford, UK; 7 Department of Pediatric Pharmacology and Pharmacogenetics, Hôpital Robert Debré, Paris, France; 8 3rd Department of Pediatrics, Aristotle University, Thessaloniki, Greece

**Keywords:** Neonate, Late-onset sepsis, Vancomycin, coagulase negative staphylococci, Randomised controlled trial, Non-inferiority, Loading dose

## Abstract

**Background:**

Vancomycin has been used in clinical practice for over 50 years; however, validated, pharmacokinetic (PK) data relating clinical outcomes to different dosing regimens in neonates are lacking. Coagulase negative staphylococci (CoNS) are the most commonly isolated organisms in neonatal, late-onset sepsis (LOS). Optimised use to maximise efficacy while minimising toxicity and resistance selection is imperative to ensure vancomycin’s continued efficacy.

**Methods:**

NeoVanc is a European, open-label, Phase IIb, randomised, controlled, non-inferiority trial comparing an optimised vancomycin regimen to a standard vancomycin regimen when treating LOS known/suspected to be caused by Gram-positive organisms (excluding *Staphylococcus aureus*) in infants aged ≤ 90 days. Three hundred infants will be recruited and randomised in a 1:1 ratio. Infants can be recruited if they have culture confirmed (a positive culture from a normally sterile site and at least one clinical/laboratory criterion) or clinical sepsis (presence of any ≥ 3 clinical/laboratory criteria) in the 24 h before randomisation.

The optimised regimen consists of a vancomycin loading dose (25 mg/kg) followed by 5 ± 1 days of 15 mg/kg q12h or q8h, dependent on postmenstrual age (PMA). The standard regimen is a 10 ± 2 day vancomycin course at 15 mg/kg q24h, q12h or q8h, dependent on PMA.

The primary endpoint is a successful outcome at the test of cure visit (10 ± 1 days after the end of vancomycin therapy). A successful outcome consists of the patient being alive, having successfully completed study vancomycin therapy and having not had a clinical/microbiological relapse/new infection requiring treatment with vancomycin or other anti-staphylococcal antibiotic for > 24 h.

Secondary endpoints include clinical/microbiological relapse/new infection at the short-term follow-up visit (30 ± 5 days after the initiation of vancomycin), evaluation of safety (renal/hearing), vancomycin PK and assessment of a host biomarker panel over the course of vancomycin therapy.

**Discussion:**

Based on previous pre-clinical data and a large meta-analysis of neonatal, PK/pharmacodynamic data, NeoVanc was set up to provide evidence on whether a loading dose followed by a short vancomycin course is non-inferior, regarding efficacy, when compared to a standard, longer course. If non-inferiority is demonstrated, this would support adoption of the optimised regimen as a way of safely reducing vancomycin exposure when treating neonatal, Gram-positive LOS.

**Trial registration:**

ClinicalTrials.gov, NCT02790996. Registered on 7 April 2016.

EudraCT, 2015–000203-89. Entered on 18 July 2016.

## Background

### Neonatal late-onset sepsis

Neonatal late-onset sepsis (LOS) is defined as sepsis occurring 48–72 h after birth. The causative organisms associated with neonatal LOS tend to be those found in the neonatal intensive care unit (NICU) or are normal skin or gut commensals. Coagulase negative staphylococci (CoNS) are the most commonly recovered organisms from the blood cultures of neonates with LOS. Overall mortality rates secondary to CoNS are reported as < 2% in resource-rich settings; however, this increases to ~ 9% in very low birth weight (VLBW) and preterm infants [1–4]. CoNS-associated LOS has a significant morbidity with episodes of LOS having an adverse effect on neurodevelopmental outcomes as well as increasing the cost of hospitalisation, primarily due to prolonged length of stay [1, 5–8]. Interventions on the NICU are often invasive, e.g. central venous catheters, and these can increase a neonate’s exposure to hospital-acquired pathogens such as CoNS [1].

### Diagnosis of neonatal sepsis

Diagnosing neonatal sepsis can be difficult, particularly as clinical signs of sepsis can frequently overlap with disorders unrelated to sepsis [9]. Blood culture positivity rates in neonates are low [10] and pharmacodynamic (PD) markers, such as C-reactive protein (CRP) can demonstrate variable results in neonates, particularly preterm and VLBW babies [11, 12]. Novel methods for diagnosing neonatal sepsis such as biomarkers based on host RNA are under development. A prospective, case-control study provided clinically validated evidence that host RNA from whole blood can serve as a causally relevant biomarker for detecting neonatal infection [13, 14].

### Vancomycin use and emerging antimicrobial resistance

Glycopeptides are the most commonly prescribed antibiotics in children for hospital-acquired infections in Latin America, North America, Australia and Europe, with vancomycin being one of the most widely utilised antibiotics in the treatment of Gram-positive sepsis. Methicillin and gentamicin resistances in CoNS are reported as 85% and in the range of 32%-94%, respectively [15–18]; vancomycin is, therefore, a first-line therapy in sepsis due to CoNS [19].

It is well-known that high rates of antibiotic use are associated with antimicrobial resistance [20–22]. The wide variation in vancomycin dosing and monitoring practices seen across NICUs demonstrates that optimal dosing and monitoring regimens are not well standardised in this population [23] and highly variable between countries and centres [24]. One UK study revealed 17 different combinations of dose, timing of dose and timing of monitoring [25]. Current vancomycin-dosing regimens recommended for preterm neonates, such as those in the European Society for Paediatric Infectious Diseases Manual of Childhood Infection – The Blue Book and the British National Formulary for Children, are based on expert opinion or on studies including only a small number of babies rather than on data generated from clinical trials [26–28].

The wider impact of different vancomycin-dosing regimens and course durations on normal, neonatal, gut and skin flora are not known. However, antibiotic use can lead to the disruption of the microbiome leading to colonisation with resistant bacteria and candida [5, 29, 30]. Colonisation with vancomycin-resistant enterococci and candida are known risk factors for invasive infection with associated morbidity and mortality [31, 32].

### Trial concept

In view of the lack of validated, neonatal, vancomycin pharmacokinetic (PK), safety and efficacy data to support optimal use, the European Medicines Agency (EMA) included vancomycin on its ‘Revised priority list for studies into off-patent paediatric medicinal products’. The EMA called for more data on the optimal dosing and monitoring regimens for preterm and term neonates with sepsis caused by staphylococci and non-pyogenic streptococci [33]. It was advised that new data should be generated from PK/PD and safety-based efficacy studies. The NeoVanc project has been developed in direct response as a planned drug discovery programme. The discovery and licencing of new antibiotics is a lengthy process; therefore, ascertaining optimal dosing of the older, off-patent vancomycin is essential to ensure the continued efficacy of this important antibiotic in the treatment of neonatal LOS [34].

## Methods and trial design

### Objectives

The primary objective of the NeoVanc trial is to compare the efficacy of an optimised vancomycin-dosing regimen to a standard vancomycin-dosing regimen in patients with LOS, known or suspected to be caused by Gram-positive microorganisms.

The Standard Protocol Items: Recommendations for Interventional Trials (SPIRIT) checklist is provided in Additional file 1.

### Study design and selection of optimised dosing regimen

The NeoVanc clinical trial (www.neovanc.org) is an open-label, European, multi-centre, Phase IIb, randomised, active control, parallel group, non-inferiority trial. A total of 300 participants are expected to be enrolled from approximately 20 NICUs in five European Union countries (Estonia, Greece, Italy, Spain and the United Kingdom).

The NeoVanc project forms part of a Paediatric Investigation Plan (PIP) which has received a favourable opinion from the EMA Paediatric Committee (PDCO). The clinical trial, as part of the PIP, aims to provide data on dosing, efficacy and safety of vancomycin in neonates, which will lead to a Paediatric-Use Marketing Authorisation (PUMA) application.

**Fig. 1:**
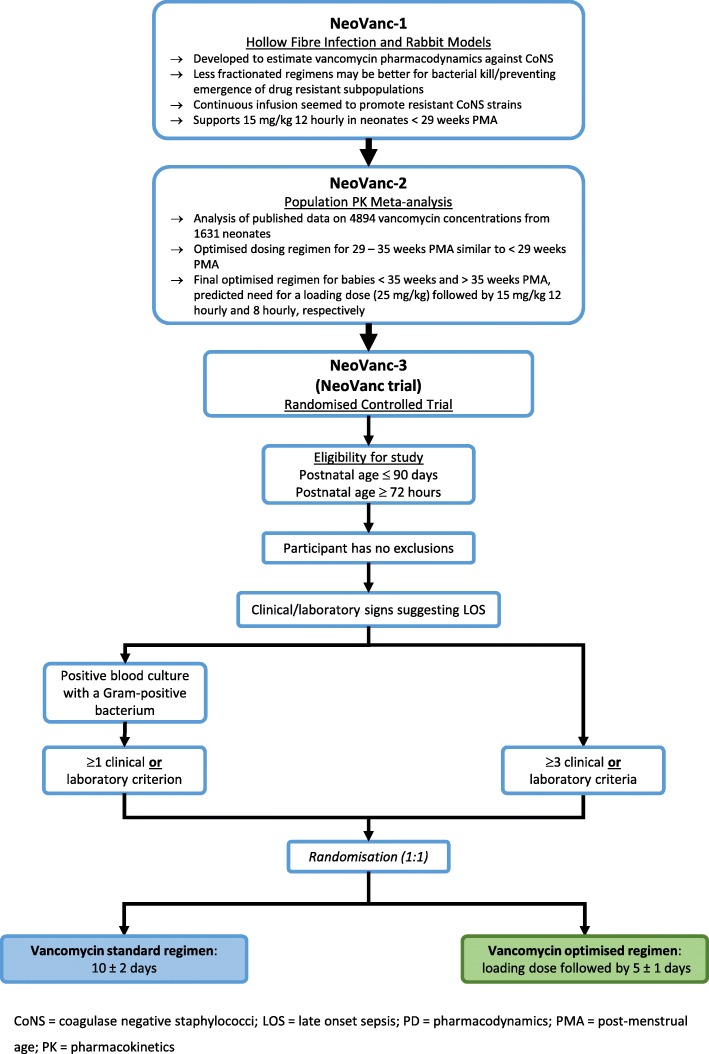
NeoVanc flow chart demonstrating how the pre-clinical studies, NeoVanc-1 (hollow fibre infection and rabbit models) and NeoVanc-2 (population pharmacokinetics meta-analysis) advised the dosing for the optimised arm of the NeoVanc Clinical Trial (NeoVanc-3)

The pre-clinical studies—(1) a hollow-fibre infection model, a rabbit model [35] and a staphylococcal biofilm *in vitro* model [36]; and (2) a population pharmacokinetic meta-analysis of neonatal, vancomycin studies [37] to define the optimal dosing regimen for the clinical trial—are detailed in Fig. 1.

Ethical approval has been gained in all participating European countries as well as approvals from the relevant Regulatory and Ethics Authorities.

### Study participants

Infants admitted to the NICU with suspected/confirmed LOS. Participants can be recruited if they fulfil the inclusion criteria and have none of the exclusion criteria.

### Inclusion criteria

The inclusion criteria for the clinical trial are adapted from the European Medicines Agency definition for neonatal LOS [38].

- Postnatal age ≤ 90 days at randomisation

AND

- Postnatal age ≥ 72 h at onset of sepsis

AND

- Clinical sepsis as defined by presence of any three clinical **or** laboratory criteria from the list below, in the 24 h before randomisation

OR

- Confirmed, significant bacterial sepsis as defined by positive culture with a Gram-positive bacterium from a normally sterile site and at least one clinical **or** one laboratory criterion from the list below, in the 24 h before randomisation.

#### Clinical criteria

- Hyperthermia or hypothermia
- Hypotension or impaired peripheral perfusion or mottled skin
- Apnoea or increased oxygen requirement or increased requirement for ventilatory support
- Bradycardic episodes or tachycardia
- Worsening feeding intolerance or abdominal distension
- Lethargy or hypotonia or irritability

#### Laboratory criteria

- White blood cell (WBC) count < 4 or > 20 × 10^9^ cells/L
- Immature to total neutrophil ratio (I/T) > 0.2
- Platelet count < 100 × 10^9^/L
- CRP > 10 mg/L
- Glucose intolerance as defined by a blood glucose value > 180 mg/dL (> 10 mmol/L) when receiving normal glucose amounts (8–15 g/kg/day)
- Metabolic acidosis as defined by a base excess (BE) < − 10 mmol/L (< − 10 mEq/L) or a blood lactate value > 2 mmol/L

### Exclusion criteria

- Administration of any systemic antibiotic regimen for > 24 h before randomisation, unless the change is driven by the apparent lack of efficacy of the original regimen
- Treatment with vancomycin for ≥ 24 h at any time within 7 days of randomisation
- Known toxicity, hypersensitivity or intolerance to vancomycin
- Known acute renal impairment as defined by urinary output < 0.7 mL/kg/h for 24 h or a creatinine value ≥ 10 μmol/L (1.13 mg/dL)
- Patient receiving (or planned to receive) haemofiltration, haemodialysis, peritoneal dialysis, extracorporeal membrane oxygenation (ECMO) or cardiopulmonary bypass
- Severe congenital malformations where the infant is not expected to survive for > 3 months
- Patient known to have *S. aureus* (MSSA or MRSA) bacteraemia
- Patient with osteomyelitis, septic arthritis, urinary tract infection (UTI) or meningitis
- Patient with high suspicion of/confirmed sepsis caused by Gram-negative organisms or fungi
- Other situations where the treating physician considers a different empiric antibiotic regimen necessary
- Current participation in any other clinical study of an investigational medicinal product (IMP)

#### Post-randomisation exclusions

- Any participant found to have Gram-negative or fungal sepsis, osteomyelitis, septic arthritis, urinary tract infection, meningitis or *S. aureus* (MSSA or MRSA) bacteraemia after randomisation will be excluded from the efficacy analysis but followed up for safety.

### Dosing schedule by arm

Standard regimen: 15 mg/kg q24h (PMA < 29 weeks), q12h (PMA 29–35 weeks) or q8h PMA > 35 weeks. Duration is 10 ± 2 days. Optimised regimen: all participants receive a single loading dose of 25 mg/kg followed by a maintenance dose of 15 mg/kg q12h (PMA ≤ 35 weeks) or q8h (PMA > 35 weeks). Duration is 5 ± 1 days. Dose adjustments should only occur within the context of routine therapeutic drug monitoring. The only exception is if renal impairment develops, when study vancomycin dose may be adjusted dependent on vancomycin levels/local practice.

#### Concomitant antibiotics

Use of concomitant antibiotics during vancomycin therapy is allowed except for specific anti-staphylococcal agents (flucloxacillin, oxacillin, linezolid, tedizolid, daptomycin and teicoplanin).

### Assignment of interventions

Infants will be randomised in a 1:1 ratio to either the standard treatment regimen or the optimised treatment regimen. Randomisation will be managed via a secure web-based randomisation facility. The randomisation program will use a minimisation algorithm to ensure balance between the groups with respect to clinical centre, PMA at randomisation, and presence or absence of an umbilical catheter/central venous line.

Local investigators and participants’ parents/guardians will not be blinded to regimen allocation as this is an open label study. Investigators undertaking the biomarker and microbiological sub-analyses will be blinded to arm allocation. The trial management group and trial data analysts will be blinded to aggregate outcomes from interim analyses; unblinding will, therefore, not occur.

### Primary outcome measure

The primary outcome incorporates clinical and microbiological factors. Successful outcome at the Test of Cure (TOC) visit (10 ± 1 days after the end of actual vancomycin therapy [EVT])

- Participant is alive

and

- Successful outcome at EVT (defined as the participant is alive, there is a significant improvement in the participant’s overall, clinical status, there is microbiological resolution or presumed eradication of bacteria and no new vancomycin-susceptible pathogens potentially associated with sepsis have been identified)

and

- Participant has not had a clinically or microbiologically significant relapse or new infection, requiring treatment with vancomycin or other specific anti-staphylococcal antibiotics (flucloxacillin, oxacillin, linezolid, tedizolid, daptomycin and teicoplanin) for > 24 h within 10 days of EVT visit

### Secondary outcome measures

- *Evaluation of efficacy*
  - Clinically or microbiologically, significant relapse or new infection within 10 days of EVT, requiring treatment with any antibiotic (other than vancomycin or specific anti-staphylococcal antibiotics [flucloxacillin, oxacillin, linezolid, tedizolid, daptomycin and teicoplanin]) for > 24 h
  - Clinically significant relapse or new infection at short-term follow-up (STFU; 30 ± 5 days from initiation of study vancomycin) visit
- *Evaluation of safety by allocation group*
  - Abnormal renal function tests at the STFU visit
  - Abnormal hearing screening test
  - Comparative safety of vancomycin (related to all other parameters other than renal and hearing safety) at STFU visit
- *PK/PD evaluation*
  - PK parameters of vancomycin using population PK modelling by allocation group
  - Probability of target attainment with different study regimens
- *Microbiological evaluation*
  - Relationship between CoNS species and duration of treatment and CRP response
  - Gut, skin and mucosal colonisation by vancomycin resistant organisms at baseline, EVT and STFU visit
  - Bacterial DNA polymerase chain reaction (PCR) analysis in babies ≥ 29 weeks PMA
- *Biomarker evaluation*
  - Assessment of changes in host biomarker panel profiles [13, 14] from baseline to EVT and the relationship between host biomarker and duration of treatment

### Study procedures

#### Recruitment and consent

The parent(s)/guardian(s) of potential participants are approached by research staff to assess eligibility, provide information and obtain informed consent. Informed consent is only gained after information has been provided, questions have been answered and time has been given for consideration. If consent is gained, the informed consent form is signed by the parents/guardians before study recruitment according to the national ethical regulations of the participating countries. Parents/guardians can also pre-consent to the trial. In this case, the same procedure applies; however, parents/guardians are approached before their baby becomes unwell. If pre-consent is in place and a baby develops signs of sepsis and meets the eligibility criteria, the parents/guardians can be contacted via telephone or in person to confirm continued consent.

Consent for the trial as well as the PK and microbiological sub-analyses are gained together; information is provided in relation to all sub-analyses in the participant information sheet and informed consent form. Participants’ parents/guardians will also be asked for permission for the research team to share relevant data with people from the organisations taking part in the research or from regulatory authorities, where relevant. A copy of the informed consent form can be provided by the corresponding author on request.

### Visits

Eight visits are planned, from the neonate’s screening and randomisation to the audiology follow-up visit and will be performed as outlined in Table 1 – Schedule of Study Assessments for the NeoVanc Clinical Trial.

**Table 1:** Schedule of study assessments for the NeoVanc clinical trial

	Visit 1a Screening and randomisation visit	Visit 1b Treatment initiation visit	Visit 2 Renal function measurement visit	Visit 3 Early on treatment visit	Visit 4 ^a^ Day 5 ± 1/ End of Allocated Therapy (optimised arm)	Visit 5 End of allocated Therapy (standard arm) visit	EVT ^b^	Visit 6 Test of Cure visit	Visit 7 Short-term follow-up visit	Visit 8 Audiology follow-up visit
Timing	Day 0	Maximum of 24 h after randomisation	Between Visit 1b and Visit 3	72 ± 8 h after initiation of study vancomycin	5 ± 1 days from initiation of study vancomycin ^a^	10 ± 2 days from initiation of study vancomycin	End of primary course of vancomycin ^b^	10 ± 1 days after end of study vancomycin	30 ± 5 days from initiation of study vancomycin	Up to Day 90 from initiation of study vancomycin
Signed informed consent	X									
Medical history	X									
Adverse event reporting		X	X	X	X	X	X	X	X	X
Clinical examination	X			X	X	X	X	X	X	
Full blood count	X	(X ^c^)		X	X	X	X	**▲**		
Renal function measurements	X	(X ^c^)	X	X	X	X	X	**▲**	X	
Glucose/Lactate/Base excess	X	(X ^c^)		X	X	X	X	**▲**		
CRP	X	(X ^c^)		X	X	X	X	**▲**		
Biomarker sampling	X	(X ^c^)		X	X	X	X		X	
Blood culture	X ^d^	(X ^c^)		(X)	(X)					
Blood for bacterial DNA analysis (babies ≥ 29 weeks PMA only)	X	(X ^c^)		X					X	
PK sampling and plasma storage ^e^										
Stool/rectal swab and colonisation swab collection	X	(X ^c^)			X ^f^	X	X		X	
Vancomycin administration		X	X	X	X	X	X			
Assessment for relapse/new infection								X	X	
Auditory screening										X

X Fixed time; **▲** To be repeated if abnormal at EVT; if laboratory results are not available, the closest values existing after EVT will be considered

^a^ All participants except those who have already undergone an EVT visit

^b^ Only participants whose vancomycin has been stopped earlier or later than outlined in the protocol

^c^ If not done at Visit 1a

^d^ If a blood culture has been taken and is positive in the 24 h before randomisation, blood culture does not need to be repeated at Visit 1a or 1b. If blood culture is positive, further cultures should be taken at each subsequent visit until culture becomes negative up to and including the Visit 4. Blood cultures do not need to be repeated if the previous culture is negative unless clinically indicated. Blood cultures should be performed between TOC and STFU in cases of relapse/new infection

^e^ Ensure PK sampling performed (< 29 weeks PMA) or samples scavenged (both (< 29 weeks PMA and ≥ 29 weeks PMA groups) at the appropriate time interval

^f^ Only participants in optimised arm

*DNA* deoxyribonucleic acid, *EVT* End of actual vancomycin therapy, *PCR* polymerase chain reaction, *PK* pharmacokinetic

### Monitoring of clinical and laboratory parameters

Participants will be monitored for specific clinical signs and laboratory parameters in line with the EMA’s criteria for the diagnosis of neonatal sepsis, at day 3, day 5 ± 1 and day 10 ± 2 (standard arm only) [38], so as to determine the rate of recovery. At EVT, an assessment will be made according to specified criteria as to whether the baby has had a significant improvement in their overall, clinical status. Participants who have met this criteria will proceed to the TOC visit where an assessment to determine whether the baby has had a clinically significant new infection (three clinical or laboratory signs), microbiological relapse (positive blood culture with phenotypically similar organism + one clinical/laboratory sign) or microbiological new infection (positive blood culture with an organism of interest + one clinical/laboratory sign) which has required > 24 h of anti-staphylococcal antibiotics since the EVT visit. All participants will undergo hearing screening.

### Study-specific assessments

#### Blood isolates

Blood culture will be collected at baseline and subsequently if positive. Isolates will be stored and sent to the central microbiology laboratory for identification and antimicrobial susceptibility testing. Relevant isolates will be tested for biofilm production and biofilm susceptibility.

#### Bacterial DNA PCR

Bacterial DNA PCR will be utilised in the identification of causative organisms where the blood culture is negative, as well as quantitative analysis as a PD marker. Bacterial DNA analysis will only take place in those infants who are ≥ 29 weeks PMA in view of the restrictions on the blood volumes allowed in clinical trials. Samples are taken at baseline, Visit 3 and the STFU visit.

#### Disruption of normal colonising flora

Stool (or rectal swab if stool unavailable) as well as axilla and nasal swabs will be collected at baseline, EVT and at the STFU visit to assess the impact of vancomycin on gut, skin and mucosal flora. Babies will be screened for vancomycin-resistant enterococci, candida and a subset for CoNS with reduced susceptibility to vancomycin, which will all be used as surrogate markers of disruption to normal flora. It will be determined whether colonisation with these organisms changes over the treatment course, as well as between specific groups, e.g. preterm versus term babies.

#### Biomarkers

A set of biomarkers will be performed as a classifier with high accuracy in predicting specifically bacterial infection. Biomarkers samples are taken at baseline, visits 3, 4, 5 (standard arm only) and the STFU visit.

### Sample size

This is a non-inferiority study comparing a standard regimen and an optimised regimen. It is hypothesised that 95% of the participants in the standard treatment arm will not require a second course of treatment with anti-staphylococcal antibiotics and that the proportion will be the same in the optimised treatment group. Using the Wilson-score method, a sample size of 150 babies per arm will give at least 90% of power to demonstrate a non-inferiority margin of 10% (nQuery Software, ‘Statsols’; Statistical Solutions Ltd., Cork, Ireland).

### Statistical analysis

Analysis of the primary outcome will be performed using binomial regression with an identity link to report the risk difference and its 95% confidence interval (CI) (risk of a successful outcome at TOC visit in the optimised regimen – risk in the standard regimen). The optimised regimen will be considered non-inferior if the lower limit of the 95% CI is ≥ − 10%. Non-inferiority of the optimised regimen will be tested using a one-sided test at the 2.5% level of significance.

An unadjusted analysis will be carried out as well as an adjusted analysis, adjusting for the minimisation factors used during randomisation by including them as covariates – centre, PMA at randomisation (< 29 weeks/29–35 weeks/> 35 weeks), and presence or absence of an umbilical catheter/central venous line. If there are insufficient participants in some centres to use this as a covariate factor, the centre will be used to adjust the variance estimator by including it as a clustering factor. This analysis will be carried out using both per-protocol (PP) and intention-to-treat (ITT) populations for comparison but the primary inference will be based on the adjusted analysis of the PP population.

For the secondary outcomes, risk ratios and their 95% CIs will be estimated using a log binomial regression model or using a log Poisson regression model with a robust variance estimator if the binomial model fails to converge. The analyses will be carried out for both the PP and ITT populations and, in each case, an unadjusted and adjusted analysis will be carried out as described for the primary outcome.

Pre-specified subgroup analysis will be performed for the primary outcome using the statistical test of interaction, based on PMA at randomisation, birth weight category, and presence or absence of an umbilical catheter/central venous line at the onset of sepsis.

### Pharmacokinetics analysis

Pharmacokinetic data will be analysed by nonlinear mixed-effect modelling method (NONMEM). The vancomycin plasma concentration data will be imported into R for exploratory analysis and formatted for subsequent modelling using NONMEM version 6.2 (Globomax, USA). An interim PK analysis will be performed when PK and scavenged samples have been obtained from 50 participants.

### Adverse event reporting

All adverse events occurring from the administration of the first dose of study vancomycin to the final follow-up visit will be recorded. In addition, serious adverse events, which meet the criteria for expedited reporting, will be reported to the Sponsor within 24 h of the awareness of the event.

### Data management

Data will be collected in an electronic case report form which will be managed by Consorzio per Valutazioni Biologiche e Farmacologiche. All data collected will remain strictly confidential and anonymous. Access to the data is mandated by the Sponsor.

### End of trial

The end of trial will be the point of database lockdown.

### Committees

Network Management Group (NMG) is an executive body comprising of NeoVanc Work Package leaders and the Trial Statistician with decision powers over the whole NeoVanc project, its technical development and work plan updates. The NeoVanc Trial Management Group is a subgroup of the NMG and comprises of the individuals actively involved in this clinical trial. The group is responsible for managing the operational aspects of the trial. The Independent Data Monitoring Committee (IDMC) will monitor progress, safety and PK data with the aim of protecting the safety and interests of the trial participants and scientific integrity of the trial. The IDMC is composed of a neonatologist, a microbiologist and a statistician. The members of the IDMC are independent and have no involvement with the NeoVanc clinical trial.

### Monitoring

Monitoring of the study will be performed by Therakind Ltd. in accordance with the International Conference on Harmonisation of Technical Requirements for Registration of Pharmaceuticals for Human Use Good Clinical Practice guidelines, local regulations and Therakind Standard Operating Procedures.

Audit can be performed at any time by independent, properly authorised individuals mandated by the Sponsor. The audit is aimed to ensure the quality of the research, data accuracy, and respect of laws and regulations.

### Specific considerations

Study-specific procedures have been kept to as few as possible and the volumes of blood to be obtained fall well within the guidelines set out by the EMA [39]. Bacterial DNA analysis will only be undertaken in neonates ≥ 29 weeks PMA. Study procedures will be carried out at the same time as routine investigations, where possible, to minimise discomfort.

Neonatal sepsis is serious and requires rapid treatment initiation; therefore, it will not always be possible to gain consent for the study within this timeframe. Up to 24 h of antibiotic administration prior to randomisation has been allowed within the protocol to allow for this. However, pre-consent is also allowed where consent can be gained before a baby becoming unwell, allowing for IMP to be initiated as soon as possible.

### Trial sponsor

The trial sponsor is Fondazione PENTA Onlus (Email: info@penta-id.org). The sponsor has not participated in the study design and will not participate in the collection, analysis or interpretation of the data.

## Discussion

### Clinical trials and neonates

The NeoVanc trial opted to adapt the EMA criteria for the diagnosis for neonatal sepsis, by allowing entry to the trial on three clinical or laboratory signs as opposed to requiring two clinical and two laboratory signs. The reason for this was due to the less severe presentation and milder sepsis course seen with CoNS [1–4].

The NeoVanc trial is an open-label study. The use of a placebo in neonatal trials is controversial particularly if preterm infants are included in recruitment. In studies evaluating intravenous medication, the use of placebo can be a challenge in view of the careful fluid balance required in this population. Blinding would be difficult in view of the differing course lengths unless a placebo was used for the remaining days in the short course arm. It is recognised by the NeoVanc Consortium that this could lead to ascertainment bias particularly in view of the varying prescribing practices for vancomycin throughout Europe; some clinicians may favour a short course over a long course or vice versa [23, 25]. Investigators will be strongly encouraged to adhere to the allocated vancomycin duration and an assessment of the baby’s clinical status will be undertaken on the day vancomycin is actually stopped as well as on the day vancomycin should have been terminated so as to ascertain if the baby fulfilled the protocol’s definition of recovered/significantly improved.

The NeoVanc trial provides the unique opportunity to evaluate multiple aspects of neonatal, vancomycin pharmacokinetics within one study. Different course lengths will be explored but also the use of a loading dose, in the optimised arm, will help to ascertain if vancomycin levels can be safely increased more quickly and thus allow course duration to be reduced. More frequent dosing in babies < 29 weeks PMA will also be assessed in the optimised arm where babies receive dosing every 12 h instead of every 24 h as in the standard arm.

Finding an acceptable balance between antibiotic resistance, safety, duration and efficacy is a major challenge when treating neonatal sepsis; the NeoVanc trial aims to focus on all four components.

## Trial status

The trial opened to recruitment on 3 March 2017; the first participant was recruited on 17 March 2017. The current protocol version is 6.0 (dated 7 September 2016). Recruitment is expected to complete by 31 December 2019.

### Dissemination policy

There are no current plans for granting public access to participant-level datasets or statistical code. Outcomes of this trial will be published in a peer-reviewed journal.

## Supplementary information

**Additional file 1.** SPIRIT 2013 Checklist.

## Data Availability

Data relating to the NeoVanc preclinical studies are included in these published articles [35–37]. No data relating to the NeoVanc clinical trial is presented in this manuscript.

## Abbreviations

CI: Confidence interval
CoNS: Coagulase negative staphylococci
CRP: C-reactive protein
CVBF: Consorzio per Valutazioni Biologiche e Farmacologiche
ECMO: Extracorporeal membrane oxygenation
EMA: European Medicines Agency
EOAT: End of allocated vancomycin therapy
EVT: End of actual vancomycin therapy
IDMC: Independent Data Monitoring Committee
IMP: Investigational Medicinal Product
ITT: Intention to treat
LOS: Late onset sepsis
NICU: Neonatal intensive care unit
NIHR: National Institute for Health Research
NMG: Network Management Group
PCR: Polymerase chain reaction
PD: Pharmacodynamics
PDCO: EMA Paediatric Committee
PIP: Paediatric Investigation Plan
PK: Pharmacokinetics
PMA: Postmenstrual age
PP: Per protocol
STFU: Short-term follow-up
TOC: Test of cure
UTI: Urinary tract infection
WBC: White blood cells
